# Acute retinal pigment epitheliitis: a case presentation and literature review

**DOI:** 10.5935/0004-2749.20210028

**Published:** 2025-02-02

**Authors:** Raşit Kılıç

**Affiliations:** 1 Department of Ophthalmology, Faculty of Medicine, Ahi Evran University, Kırşehir, Turkey

**Keywords:** Retinal diseases, Retinitis pigmentosa, Epithelium, Retinal pigments, Circadian clocks, Retinal photoreceptor cell outer segment, C-mer tyrosine kinase, Visual acuity, Doenças retinianas, Retinite pigmentosa, Epitélio, Pigmentos retinianos, Relógios circadianos, Segmento externo das células fotorreceptoras da retina, C-mer tirosina quinase, Acuidade visual

## Abstract

Acute retinal pigment epitheliitis (ARPE) is an idiopathic, self-limiting inflammatory retinal disorder that particularly affects healthy young individuals. The characteristic fundoscopic appearance of the acute retinal pigment epitheliitis includes a fine pigment stippling surrounded by a yellow-white hypopigmented halos in the macula. Although the exact pathogenesis of the disease remains unknown, some reports have suggested a relationship between a viral infection and acute retinal pigment epitheliitis. Acute retinal pigment epitheliitis is a rare disorder, and only single case reports or case series are found in the literature. The clinical and demographic characteristics of patients with this disease are not fully understood because of its rarity. In this study, we searched the literature to collect clinical and demographic features of the reported cases. We detail the characteristics of acute retinal pigment epitheliitis were pointed and discuss the pathogenesis of the disease.

## Abstract (pt)

A epitelite pigmentar retiniana aguda (EPRA) é uma doença inflamatória idiopática e autolimitada da retina, que afeta especialmente indivíduos jovens e saudáveis. À fundoscopia, a aparência característica dessa entidade é de um pontilhado fino do pigmento, cercado de halos hiperpigmentados branco-amarelados na mácula. A patogênese exata da doença ainda é desconhecida, mas alguns relatos apontam uma relação entre epitelite pigmentar retiniana aguda e infecções virais. A epitelite pigmentar retiniana aguda é uma condição rara e na literatura há apenas relatos de casos individuais ou séries de casos. As características clínicas e demográficas da doença não são totalmente compreendidas, devido à sua raridade. Para este relato, foi feita uma busca na literatura para coletar os dados clínicos e demográficos dos casos relatados. Finalmente, são apontadas as características da epitelite pigmentar retiniana aguda e discute-se a patogênese da doença.

## INTRODUCTION

Acute retinal pigment epitheliitis (ARPE) was first described by Krill and Deutman in 1972^(1)^. It is a rare, idiopathic, and self-limiting inflammatory retinal disorder that affects healthy young individuals in particular. The characteristic fundoscopic appearance of ARPE is a fine pigment stippling surrounded by a yellow-white hypopigmented halo in the macula^(2)^. Patients generally suffer from blurred vision, central scotoma, and metamorphopsia^(3)^. Although the exact pathophysiological pathway of the disease remains unknown, some reports have suggested a possible relationship between a viral infection and ARPE^(4)^.

The first reports of ARPE described the primary inflammation area as the retinal pigment epithelium (RPE). Currently, high-resolution optical coherence tomography (OCT) is able to provide in vivo cross-sectional images from the retina, and OCT findings have revealed that the primary inflammation area is in the outer retinal layers, especially in the interdigitation zone (IZ), ellipsoid zone (EZ), and external limiting membrane (ELM), rather than the RPE^(2,4-15)^

ARPE is a rare disorder, and the relevant literature includes only single case reports or case series. The clinical and demographic characteristics of patients with the disease are not fully understood because of its rarity. In this study, we searched the literature to find case reports and collect clinical and demographic features in patients with ARPE. We also present an ARPE case in this review. Finally, we detail the characteristic features of the disease and discuss its pathogenesis.

## METHODS

We searched the literature using PubMed to find ARPE case reports. We used the search terms “acute retinal pigment epitheliitis”, “acute retinal pigment epithelitis”, “acute retinal pigment epitheliitus” and “retinal pigment epitheliitis” to identify related articles in PubMed. A total of 38 articles were found; however, full texts of only 29 articles were available. Cases that underwent either fluorescein angiography (FA) or OCT imaging during the diagnosis were included in the review^(1-22)^. We identified a total of 72 cases among the 29 full-text articles. We excluded eight cases that had atypical fundoscopic and angiographic appearance, two cases that were not in the acute phase of the disease, and two cases that had no FA or OCT imaging findings. Ultimately, a total of 60 cases were included. When including the present case, a total of 61 patients with 67 involved eyes were evaluated in this review.

## RESULTS

### Case presentation

**Figure 1:**
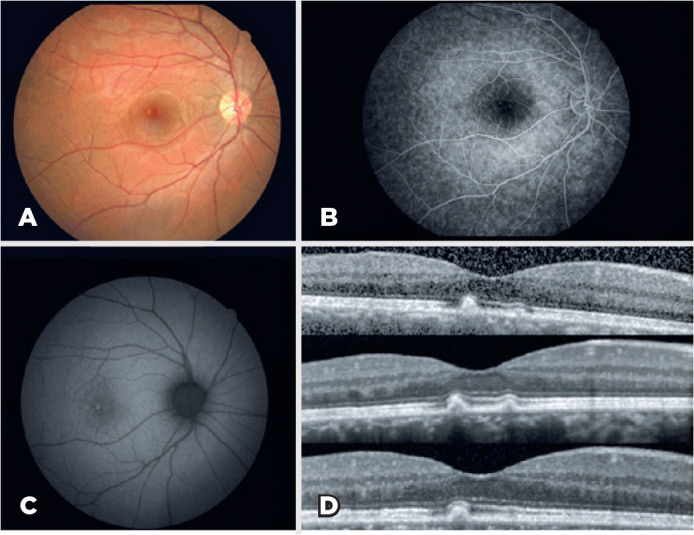
A) Fundus color photography shows a localized pigment stippling area associated with yellowish hypopigmentation in the fovea. B) fluorescein angiography shows transmission hyperfluorescence without leakage in the fovea. C) Fundus autofluorescence imaging shows hyperautofluorescence in the lesion area. D) Upper optical coherence tomography image shows abnormal hyperreflectivity in the EZ and RPE area upon patient presentation. Middle and bottom images were obtained at one-and six-month follow-up, respectively.

A 35-year-old male patient presented to our clinic with a metamorphopsia in his right eye for one day. His ocular history and systemic medical history were unremarkable. There was also no history of viral infection. Visual acuity was 20/20 in both eyes. The anterior segment examination using a slit-lamp was normal. Fundus examination revealed a localized pigment stippling area associated with yellowish hypopigmentation in the fovea. Transmission hyperfluorescence without leakage was observed upon FA. Fundus autofluorescence (FAF) revealed an increased autofluorescence in the lesional area. OCT showed abnormal hyperreflectivity in the EZ and RPE areas (Figure 1). Metamorphopsia disappeared after one week. OCT and FAF findings remained unchanged at one-, three-, and six-month follow-up. However, the patient’s metamorphopsia and visual symptoms had resolved.

### Demographic and clinical features of ARPE

It is well known that ARPE affects healthy young individuals, with the mean patient age being 30.6 ± 10.7 years (Table 1). However, no data have been reported regarding gender distribution. In this review, we found a female predominance of 62.3%, approximately two-thirds of all the cases included. A total of 58 case presentations had a clear medical history, and a relationship between viral infection and ARPE was detected in 15 (25.9%) cases. While the right eye was affected in 28 (57.1%) cases, the left eye was affected in 21 (42.9%) cases. Six patients^(3,5-8,16)^ (9.8%) had bilateral involvement, and two cases^(3,9)^ were recurrent. Findings of popper’s syndrome are similar to those of ARPE, and it is generally seen in both eyes. We found popper use query in only one case presentation from six bilaterally cases, and it was negative^(5)^. The median visual acuity was 20/40 (0.32 ± 0.20 logMAR) at the first examination, which reached 20/20 (0.03 ± 0.09 logMAR) at the final follow-up.

**Table 1:** Demographic and clinical features of acute retinal pigment epitheliitis cases.

Age (years)	30.6 ± 10.7 (16-55)	
Gender (male/female)	23 (37.7%)/38 (62.3%)	
Symptom duration (days)	7.5 ± 6.1 (1-30)	
Initial visual acuity	0.32 ± 0.20 (0-0.9)	
Final visual acuity	0.03 ± 0.09 (-0.1 to 0.5)	
Eye laterality (right/left)	28 (57.1%)/21 (42.9%)	
Viral infection history (absent/present)	43 (74.1%)/15 (25.9%)	
Affected retinal layers (*n*=45 eyes)	Interdigitation zone	45 (100%)
	Ellipsoid zone	43 (95.6%)
	External limiting membrane	16 (35.6%)
	Outer nuclear layer	12 (26.7%)
	Retinal pigment epithelium/Bruch complex	4 (8.9%)
Fluorescein angiography changes (*n*=61 eyes)	51 (83.6%)	
Indocyanine green angiography changes (*n*=13 eyes)	12 (92.3%)	
Fundus autofluorescence imaging changes (*n*=10 eyes)	4 (40%)	
Bilaterally affected cases	6 (9.8%)	
Recurrence	2 (3%)	

### Imaging

OCT findings showed that the IZ was affected in all cases, the EZ in 43 (95.6%) cases, the ELM in 16 (35.6%) cases, and the outer nuclear layer (ONL) in 12 (26.7%) cases. RPE/Bruch’s complex was observed as mildly thickened in only four eyes (8.9%) of four cases^(2,4-15)^.

We determined that FA was carried out in 61 eyes of 56 cases^(1-7,12-22)^. In most of the eyes (83.6%), the characteristic FA finding was transmission hyperfluorescence without leakage (Table 2). Autofluorescence imaging was performed in only 10 eyes of nine cases, with a mild hyperautofluorescence observed in the lesional area in four eyes of four patients^(2,5,9,11-13)^. We observed that indocyanine green angiography (ICGA) was carried out in 13 eyes of 12 cases^(2,4,5)^. ICGA revealed a hypofluorescent lesion surrounded by a hyperfluorescent halo with a cockade-like appearance in 10 eyes of nine cases. Patchy hyperfluorescence with a hyperfluorescent halo was observed in one eye, whereas patchy hyperfluorescence alone was detected in one eye. One eye showed no significant finding in the fovea.

**Table 2:** Diagnostic features of the acute retinal pigment epitheliitis.

Fundus appearance • Localized pigment stippling areas associated with yellowish, halo-like zones of hypopigmentation in the fovea • No finding of any other ocular disorder
Optical coherence tomography
• 1Z is affected in all cases
• EZ is affected in 95.6% of eyes
Fluorescein angiography
• Transmission hyperfluorescence without leakage in the fovea is seen in
83.6% of cases
Indocyanine green angiography
• Hyperfluorescence is seen in 92.3% of eyes
• Hyperfluorescent halo with a cockade-like characteristic appearance is
found in 84.6% of cases

Only 11 case reports were found that addressed the healing duration of the retinal layers^(2,5,8,11,12,15)^. According to the reports, both the IZ and EZ were affected in all cases, whereas the ELM was affected in only seven cases. The mean durations of healing in the ELM, EZ, and IZ were 5.1 ± 4.8, 7.2 ± 5.2, and 9.1 ± 8.3 weeks, respectively. The results suggest that the disease heals itself. On the other hand, we found that in two cases, the persistent disruption of EZ did not recover after more than 12 months of follow-up.

## DISCUSSION

### Pathogenesis of ARPE

Photoreceptors and other retinal neurons are sensitive to alternations of night and day and to seasonal changes. Retinal signals convey information about temporal changes to a central clock located in the suprachiasmatic nucleus of the hypothalamus. Thus, the retina informs the brain about light changes and provides core information for the central circadian clock, which arranges the biological rhythms to environmental light-dark cycles^(23)^. The physiological functions of the retina are also under the control of an internal retinal circadian clock, including circadian clock gene expression, dopamine synthesis, melatonin release, gamma-aminobutyric acid release, photoreceptor disk shedding, and retinal sensitivity to light^(24,25)^. The retinal circadian clock is controlled by cell-autonomous clocks, which are located in the retinal layers, especially in the inner nuclear layer^(26)^. Retinal dopamine plays an important role in the retinal circadian clock, which rearranges retinal circulation to enhance cone-mediated visual signaling during photopic conditions and rod-mediated visual signaling at scotopic conditions for the adaptation of visual sensitivity to the temporal alternations in the natural environment^(25,26)^. Disruption of the circadian clocks may have some effects on many disorders, including retinal degeneration and glaucoma^(26)^.

One of RPE’s essential functions is its contribution to the renewal of the photoreceptor outer segment for both rods and cones^(26-28)^. The renewal of the photoreceptor outer segments in the retina includes the continuous synthesis of new membranous disks at the proximal end and circadian disc shedding at the distal tips. Rapid and complete phagocytosis of spent photoreceptor outer segment fragments (POS) by RPE is vital for photoreceptor survival/function and vision^(27,28)^. RPE phagocytosis of spent POS occurs in three phases: recognition and binding, engulfment, and lysosomal digestion^(27-30)^. The first phase that is the recognition and binding of POS occurs via αvβ5 integrin. The physiological ligand of αvβ5 integrin is the secreted glycoprotein milk fat globule-E8 (MFG-E8) during the first phase^(29)^. At the second phase, attachment of αvβ5 receptors by MFG-E8 and POS leads to two independent signals that activate Mer tyrosine kinase (MerTK) via focal adhesion kinase and Rac1 GTPase. Both signaling pathways are essential for the engulfment of spent POS^(29,30)^. Finally, the engulfed POS is removed by lysosomal digestion within the RPE cells. Circadian rhythms regulate rod and cone POS renewal, and they have similar processes; however, their turnover of POS differs markedly in their diurnal rhythms. Although circadian cone POS shedding occurs with the onset of night, rod POS shedding happens in the morning with light onset^(29,30)^.

Daily phagocytosis of spent POS by RPE cells is crucial for vision. Even an insufficiency or delay in spent POS clearance leads to an accumulation of photoreceptor fragments. A lack of αvβ5 integrin receptor results in age-related accumulation of outer segment remnants^(30,31)^. However, overexpression of αvβ5 receptors has little effect on spent POS binding^(27)^.

MerTK is a negative regulator of αvβ5 integrin-dependent POS binding, and its deficiency results in excess POS binding by RPE cells^(27)^. In animal models, MerTK deficiency and lack of engulfment cause dramatic, fast, and complete retinal degeneration. It has also been shown that MerTK mutations lead to retinitis pigmentosa in humans^(30)^.

OCT images show a hyperreflectivity in the IZ, EZ, ELM, and ONL with different frequencies in ARPE cases. This hyperreflectivity may be related to undigested, spent POS accumulation. We therefore concluded that the pathogenesis of ARPE may be, in the second phase, spent POS clearance. Overexpression of the αvβ5 integrin receptor has limited effects on spent POS binding. On the other hand, MerTK deficiency causes a dramatically increased spent POS binding with a lack of engulfment. Therefore, MerTK deficiency may be at the center of ARPE pathogenesis. Puche et al also reported that the pathogenesis of ARPE may be related to MerTK deficiency^(32)^. Consequently, it can be suggested that acute and transient MerTK deficiency may lead to a dramatically spent POS accumulation in the outer retinal layers. Increased spent POS may disrupt the outer retinal layers and cause photoreceptor cell loss in severe cases. However, the trigger factor of the disease remains unclear. Viral infection is observed in 25.9% cases, and this may be a trigger factor in some patients. Further studies should investigate this interpretation to fully understand the pathogenesis of ARPE.

ARPE is a rare and self-limiting disorder that can self-recover within 6-12 weeks without treatment^(1)^. However, there remains a lack of information about sex distribution, eye laterality, relationship with viral infection, clinical characteristics, and the natural course of the disease. Therefore, in this review, we collected data from the relevant literature to improve knowledge about ARPE. Based on the data we collected, we found a female predominance, and most of the cases showed unilateral involvement. An excellent visual prognosis was also observed. There were characteristic findings on fundoscopic examination, OCT, FA, FAF, and ICGA. We also discussed the potential pathophysiological mechanisms with the aim of contributing to the understanding of the pathogenesis. The IZ was pointed out as the main area of the disease. All of this information, taken together, may be useful during the diagnosis of the disease and in the understanding of its pathogenesis.
